# Ligation of the Tibilias Anticus Artery

**Published:** 1859-02

**Authors:** A. Heavenridge

**Affiliations:** Stilesville, Ind.


### ARTICLE VI.

### LIGATION OF THE TIBIALIS ANTICUS ARTERY.

BY A. HEAVENRIDGE, M.D., STILESVILLE, IND.

*June* 11*th*, 4 *o'clock P. M.*—Called to see E. Bridgwaters, a boy nine years old. Found him with an incised wound, three inches long, in the dorsum of the foot, commencing at the extremity of the metatarsal bone of the great toe, and running parallel with it, dividing the dorsopedalis artery, and some of its communicating branches, and the plantar arteries. The wound had been received five weeks before, at which time it was dressed by my friend Dr. Gibbons, but the arteries, not being ligated, continued to effuse blood from time to time, until at the time I saw him, when he was completely exsanguine. I dressed the wound in such a way, using compresses and styptics, as to prevent hemorrhage for the time being, until I could procure assistance, intending to ligate.

*June 12th, 9 o'clock A. M.*—Saw the patient in connection with Drs. Matthews and Green. After some deliberation, Dr. Matthews suggested the use of a compress on the dorsum of the foot, a little above the incision, where the artery could be seen pulsating, which was agreed to by Dr. Green and myself, and applied. The foot being swollen at the time, the compress soon became loose, in consequence of the swelling subsiding, and hemorrhage again occurred, after a period of nine days, when I was again summoned to see him in great haste. When I arrived the blood had stopped running, in consequence of a coagulum that had formed in some lint that had been placed around the bleeding orifice. On removing the clot, the hemorrhage was truly alarming. I again applied compresses and styptics, until I could procure assistance to ligate.

*June 22d, 9 o'clock A. M.*—I saw the patient in company with Dr. Green. There had been no bleeding during the night. After putting him under the influence of chloroform, we cut down on and tied the artery. There still remained a small branch of the plantar arteries, communicating with the wound, which conveyed some blood. Not being able to ligate, I cauterized it with nitrate of silver, and dressed the wound, giving the patient a quarter of a grain of the sulphate of morphia before leaving him.

*June 24th.*—The foot was badly swollen and painful; rested very poorly during succeeding twenty-four hours after operation.

*June 28th.*—The swelling gone down; wound where the artery was ligated almost filled up; old wound doing well. Constitutional condition good, excepting the extreme anemia.

*July 5th.*—The foot doing very well. The wounds are almost healed up; appetite good, which had been rather poor before, and at times almost complete anorexia.

*July* 10*th*.—Wounds completely filled, though tender yet; appetite good, bowels regular. Mucous membranes have resumed a healthy color, whereas they were of a sallow white. The pulse is soft and natural, which was very quick and irritable during his extreme anemia.

The constitutional treatment in this case has been a liberal use of chalybeates, with a small portion of vegetable tonics, and as occasion required, opiates and laxatives. The object that I wish to attain in reporting this case, is not merely to show that the artery was ligated successfully seven weeks after the wound was received, and after the patient was reduced to the very extreme of anemia, but to give my professional brethren, who may read the *Journal*, and are like myself, mere novices in the healing art, a hint of the importance of looking well to the suppression of hemorrhage, before dressing and leaving a wound. Had the arteries been tied when this wound was received, the patient would have been well in half the time, and that, too, without any risk of life.

